# Probing the potential of mucus permeability to signify preterm birth risk

**DOI:** 10.1038/s41598-017-08057-z

**Published:** 2017-09-04

**Authors:** K. B. Smith-Dupont, C. E. Wagner, J. Witten, K. Conroy, H. Rudoltz, K. Pagidas, V. Snegovskikh, M. House, K. Ribbeck

**Affiliations:** 1 Department of Biological Engineering, Massachusetts Institute of Technology, Cambridge, MA 02139 USA; 2 Center for Environmental Health Sciences, Massachusetts Institute of Technology, Cambridge, MA 02139 USA; 3 Department of Mechanical Engineering, Massachusetts Institute of Technology, Cambridge, MA 02139 USA; 4 Computational and Systems Biology Initiative, Massachusetts Institute of Technology, Cambridge, MA 02139 USA; 5 Obstetrics and Gynecology, Maternal-Fetal Medicine, Tufts Medical Center, Boston, MA 02111 USA; 6 Center for Reproduction and Infertility, Women & Infants Hospital, Warren Alpert Medical School of Brown University, Providence, RI 02905 USA; 7 Maine Medical Partners Women’s Health, Maternal-Fetal Medicine, Portland, ME 04102 USA; 8 Present Address: Department of OB/GYN & Women’s Health, Reproductive Endocrinology and Infertility, University of Louisville School of Medicine, Louisville, KY 40205 USA; 9 Present Address: Center for Reproduction and Infertility, Women & Infants Hospital, Warren Alpert Medical School of Brown University, Providence, RI 02905 USA

## Abstract

Preterm birth is the leading cause of neonatal mortality, and is frequently associated with intra-amniotic infection hypothesized to arise from bacterial ascension across a dysfunctional cervical mucus plug. To study this dysfunction, we assessed the permeability of cervical mucus from non-pregnant ovulating (*n* = 20) and high- (*n* = 9) and low-risk (*n* = 16) pregnant women to probes of varying sizes and surface chemistries. We found that the motion of negatively charged, carboxylated microspheres in mucus from pregnant patients was significantly restricted compared to ovulating patients, but not significantly different between high- and low-risk pregnant women. In contrast, charged peptide probes small enough to avoid steric interactions, but sensitive to the biochemical modifications of mucus components exhibited significantly different transport profiles through mucus from high- and low-risk patients. Thus, although both microstructural rearrangements of the components of mucus as well as biochemical modifications to their adhesiveness may alter the overall permeability of the cervical mucus plug, our findings suggest that the latter mechanism plays a dominant role in the impairment of the function of this barrier during preterm birth. We expect that these probes may be readily adapted to study the mechanisms underlying disease progression on all mucosal epithelia, including those in the mouth, lungs, and gut.

## Introduction

Preterm birth, defined as birth prior to 37 weeks of gestation, affects up to 18% of pregnancies world-wide, and is the leading cause of neonatal death and the second leading cause of childhood death below the age of 5 years^1^. In addition to this immediate mortality risk, children born preterm who survive beyond infancy are predisposed to short-term complications related to the incomplete development of multiple organ systems, as well as long-term developmental disorders and early death^1, 2^. Altogether, the annual financial burden of preterm birth and its related complications is estimated at $26 billion in the United States^1^.

Although the causes of preterm birth are complex and multifaceted, intra-amniotic infection, which facilitates preterm birth by triggering an inflammatory response resulting in cervical ripening, weakening of the chorioamniotic membrane, and myometrial contractions^3^, is present in an estimated 25–40% of clinical cases^1, 2^. During healthy pregnancy, cervical mucus forms a compact, protective ‘plug’ between the sterile uterus and the colonized vagina, selectively permitting the passage of desirable agents such as nutrients, gases, and immunological factors, while excluding potentially deleterious environmental particles and pathogens^4–7^. Since one pathway for intra-amniotic infection is ascension of pathogens across the cervical mucus plug, changes in the permeability of this barrier have long been suspected to play a critical role in the etiology of preterm birth^4, 6, 7^. For instance, the presence of bacteria in amniotic fluid cultures is strongly associated with a shortened cervix, which suggests that the length of the cervical barrier is important for blocking bacterial ascension from the vagina^8^. Further, the bacteria that infect the chorioamniotic membranes in association with preterm birth are often the same species found in the vaginal flora, suggesting that ascension occurred through the cervix^3^. Indeed, every wet epithelial surface of the body is shielded by a similar mucus barrier, and it is well established in other organ systems that numerous medical conditions including asthma^9^, cystic fibrosis^9–12^, ulcerative colitis^13–15^, and chronic obstructive pulmonary disease^16^ are intimately related to local changes in mucus properties and permeability.

The primary contributor to the gel-forming properties of mucus are mucin glycoproteins, which consist of bottlebrush-like, densely glycosylated segments and bare hydrophobic regions^4–7, 17^. Mucin molecules form a viscoelastic gel capable of selectively transporting foreign molecules on the basis of size, chemical interactions, or a combination of the two^3, 4, 8, 18–22^. Consequently, modifications to the mesh size of the network or the physicochemical properties of the constituent mucin molecules would be expected to strongly influence the permeability of mucus as well as its macroscopic mechanical response^23^. In a previous study^4, 6, 7^, we showed that the viscoelastic moduli of cervical mucus from patients at high risk for preterm birth are significantly lower than those measured in samples from low-risk pregnancies, and that mucus from high-risk pregnancies exhibits a significantly greater degree of spinnbarkeit under extension. Here, we build on this work and interrogate whether permeability measurements at both the micro- and nanoscales in cervical mucus are altered in women at high risk for preterm birth. We note that while the design of our study makes it difficult to causally link changes in mucus permeability and early delivery, it does permit for clear associations of our measured permeability markers with preterm labor and subsequent preterm birth.

## Results and Discussion

We employed two classes of probes to dissect the selective permeability of mucus samples: negatively charged carboxylated microspheres 1 μm in diameter, and charged positive and negative peptides <10 nm in size. Negatively charged (carboxylated) particles were selected for the larger probes as a result of previous findings of charge-mediated impaired diffusion for positively charged (amine functionalized) particles in mucus and mucin gels^9, 12, 20, 24^. Additionally, although further surface functionalization such as PEGylation has been shown to reduce particle-mucus interactions to an even greater degree than carboxylation alone^9–12, 25–27^, this effect for particles >500 nm in diameter was found to be minimal^12, 25^. Although we certainly cannot altogether rule out the possibility of mucus-particle interactions, our chosen particle length scale of 1 μm (relevant for many bacteria, sperm cells, and, as noted by Hill *et al*.^12^, large drug-delivery vehicles) is comparable to the upper reported limit of the characteristic mesh size of native cervical mucus^28–30^. As such, steric interactions with the surrounding mucin network should be the dominant mechanism for impaired diffusive motion at this length scale. Correspondingly, we hypothesized that the diffusion of the larger probes should be altered primarily by structural reorganization of the surrounding network, while the diffusion of the smaller probes, which are minimally affected by sterics, should allow us to interrogate molecular interactions between the peptide analytes and the mucus gel.

**Figure 1:**
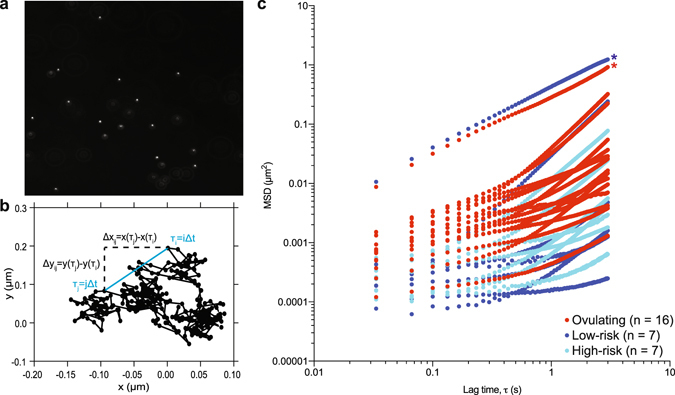
SPT of cervical mucus reveals significant differences in particle mobility between pregnant and ovulating patients but not between low- and high-risk pregnant patients. (**a**) A portion of the field of view of a single frame from a typical movie of mucus containing randomly dispersed 1 μM fluorescent particles. (**b**) Sample trajectory for a single particle from (**a**). Dots denote the *x* and *y* positions of the particle recorded by the feature-finding software at each of the 300 time points separated by time ${\rm{\Delta }}t$. The method of calculation of a single squared displacement ${\rm{\Delta }}{{r}_{ij}}^{2}$ at a lag time of ${\tau }_{ij}=(j-i){\rm{\Delta }}t$ is shown, where ${\rm{\Delta }}{{r}_{ij}}^{2}={\rm{\Delta }}{{x}_{ij}}^{2}+{\rm{\Delta }}{{y}_{ij}}^{2}$. The MSD at a given lag time for one specimen is obtained by averaging this individual displacement over those from all equivalent lag times within the trajectory of a particular particle, and then over all of the tracked particles. (**c**) MSD results as a function of lag time for all cervical mucus specimens. Dark blue symbols, low-risk pregnant patients; light blue symbols, high-risk pregnant patients; red symbols, ovulating patients. The patients with the overall highest MSDs (**c**, starred) are suspected outliers from their respective patient groups (Methods).

To assess the motion of the microspheres in mucus, we used single-particle tracking (SPT), a microrheological technique for examining the biophysical properties of complex fluids through examination of the passive motion of randomly embedded particles (Fig. 1a and b). This technique is routinely used in the design of drug-delivery vehicles, which must interact minimally with the various mucosal layers of the body in order to reach their target tissues prior to being cleared^22, 26^. The relationships that can be drawn between the thermal fluctuations of these particles and the linear viscoelastic properties of the surrounding medium^31, 32^ have led to regular use of this technique in the assessment of the mechanical properties of biological fluids including mucin-based gels, such as the effect of pH on the mesh size of native cervical mucus^33^, the effect of pH and salt on the microrheology of reconstituted MUC5AC gels^23^, and the effect of elevated mucin concentration on the elasticity of diseased airway sputum^12^. In this work we show that SPT in cervical mucus can be used to distinguish between pregnant and non-pregnant (ovulating) women, but not between women at high and low risk for preterm birth.

For normal diffusion, as occurs in a homogeneous medium with no memory (i.e. no elastic character) and with which the microspheres do not interact, the mean squared displacement (MSD) of a particle is expected to scale linearly with the lag time, and in two dimensions the explicit form for this scaling is^34^

1 $$\langle {r}^{2}(\tau )\rangle =4D\tau ,$$

where *D* is the diffusion coefficient of the microsphere in the medium. When this scaling does not hold, the diffusion is termed *anomalous*, and the MSD is often expressed in power-law form as

2 $$\langle {r}^{2}(\tau )\rangle =4{D}_{\alpha }{\tau }^{\alpha },$$

where *D*_*α*_ is a generalized diffusion coefficient and the exponent *α* can be lag-time dependent. When *α* < 1, the motion of the particle is *subdiffusive*, and when *α* >  1, the motion is superdiffusive. Anomalous diffusion has been reported in biological fluids such as lung mucus^12^, F-actin gels^35^, and plasma membranes^36^, reflecting the complex microstructure that these media generally possess.

We measured the diffusion behavior of particles in cervical mucus from 16 ovulating patients, 7 low-risk pregnant patients, and 7 high-risk pregnant patients (Table 1). The low- and high-risk pregnant patients were gestational age-matched at the time of mucus collection (*P* = 0.36, Student’s t-test), but the high-risk patients gave birth before 37 weeks of gestation (*P* = 4.29E-4, Student’s t-test). To quantify our SPT results, we fit the ensemble average MSDs as a function of lag (or delay) time τ in all of the cervical mucus samples (Fig. 1c) with power laws of the form in Eq. 2 for *τ* ≤ 0.3*s*. These early lag times were selected for fitting as a result of the observation of drift in many of the cervical mucus samples, as evidenced by the near quadratic dependence of the MSD on τ at delay times above 1 s (Fig. 1c). This drift appears to be the result of relaxation within the mucus samples; detailed discussion of our drift-correction procedures appears in the Methods. Further, we identified two outliers (starred, Fig. 1c

**Table 1:** Patient characteristics associated with samples of cervical mucus used for single particle tracking. *P* values were calculated using a two-tailed Student’s t-test.

Characteristics	Non-pregnant (n = 16)	Pregnant (n = 14)		p-value
	Ovulatory (n = 16)	Low risk (n = 7)	High risk (n = 7)	
Age (years)	35.13 (±3.44)	28.00 (±4.97)	26.83 (±6.91)	
Race (%)				
White	100.00	28.57	57.14	
Black	0.00	0.00	14.29	
Hispanic	0.00	57.14	14.29	
Other	0.00	14.29	14.29	
History (%)				
Nullipara	68.75	0.00	0.00	
Primipara	0.00	57.14	71.43	
Multipara	31.25	42.86	28.57	
Prior PTB (%)	0.00	0.00	0.00	
Gestational Age (weeks)				
Collection	NA	31.12 (±2.36)	29.84 (±2.68)	0.36
Delivery	NA	39.94 (±0.45)	34.59 (±2.12)	4.29E-04
Positive GBS (%)	NA	28.57	28.57	
Dilation (cm)	NA	0.0 (±0.0)	3.1 (±1.4)	8.24E-04

Values represented as mean (± standard deviation) or percent (%) where applicable.

), one in the ovulating group and one in the pregnant patient group, which we excluded from subsequent statistical analysis (Methods).

**Figure 2:**
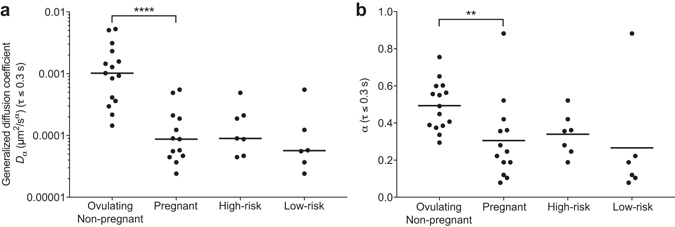
The generalized diffusion coefficient *D*_*α*_ (**a**) and the exponent *α* (**b**) extracted from the MSD results at early lag times (*τ* ≤ 0.3 s) are significantly lower in pregnant patients compared to ovulating ones, implying that the micron-sized probe particles are significantly less mobile in these samples. Pregnant patient data are further subdivided into high- and low-risk groups, but significant differences between these same two parameters are not observed at this level. Each point represents a single patient sample from non-pregnant ovulating or pregnant patients. Bars, median values for each patient group. Significance was determined with the Mann-Whitney test (****P < 0.0001; **P = 0.0021).

The mobility of the microspheres in mucus from ovulating patients (Fig. 1c, red) was notably greater than that in mucus from pregnant patients (Fig. 1c, blue). At early lag times, both the MSD exponent *α* (*P* = 2.1E-3, Mann-Whitney test) and the generalized diffusion coefficient *D*_*α*_ (*P* < 1.0E-4, Mann-Whitney test) were significantly higher in ovulating patients (Fig. 2a and b), with $\alpha =0.492$ and ${D}_{\alpha }=1.022\,\times {10}^{-3}\mu {m}^{2}/{s}^{\alpha }$ the median values for ovulatory mucus and $\,\alpha =0.247$ and ${D}_{\alpha }=8.698\,\times {10}^{-5}\mu {m}^{2}/{s}^{\alpha }$ the median values for pregnancy mucus. In addition, at early delay times *α* was greater in samples from high-risk than low-risk patients ($\alpha =0.356\,\,$and $\alpha =0.154$, respectively), although the difference was not statistically significant for this limited sample size (*P* = 7.98E-2).

The lower value of *α* at early lag times in pregnancy mucus compared to ovulatory mucus, and low-risk mucus compared to high-risk mucus, corresponds to more confined particle trajectories^31^. This confinement may arise from a possible combination of increased viscoelasticity and/or increased periods in which the probes are locally caged by their surroundings^31^. These results in ovulatory mucus are consistent with previous observations of marked structural reorganization in cervical mucus over the course of the menstrual cycle, which appear to maximize the permeability of the mucus to the passage of sperm during ovulation^37^. Taken together, these data reveal a significant association between pregnancy state and the physical microstructure of cervical mucus. However, the statistical ambiguity in the stratification of low- and high-risk samples using micron-sized probes and SPT suggests that structural reconfiguration alone cannot completely account for the changes in the permeability and viscoelasticity of cervical mucus from patients at high risk for preterm birth. As such, we next assessed the impact of mucosal function and pregnancy health on the *biochemical* properties of all of the samples of cervical mucus using peptide analytes at the greatly reduced length scale of 10 nm.

**Figure 3:**
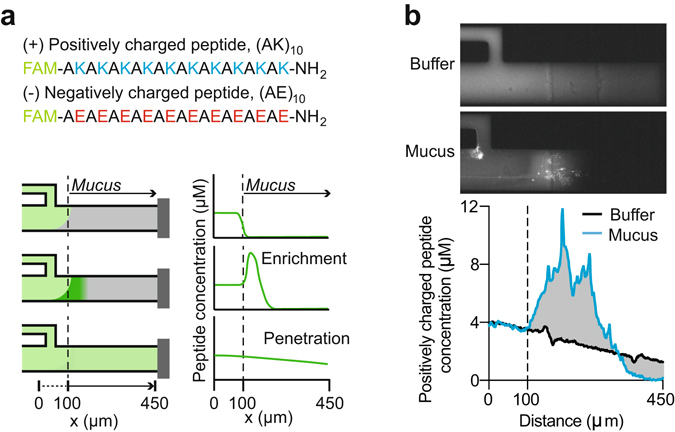
Reporter peptide sequence, microfluidic design, and quantitative analysis of mucus permeability. (**a**) Positively (+) and negatively (−) charged peptide sequences. Schematic diagrams of the microfluidic device illustrate two phenomena observed in the diffusion behavior of peptides through mucus, and the resulting concentration profiles from those behaviors. In all concentration profiles and schematics, the vertical, dotted line indicates the buffer-mucus interface. (**b**) Images obtained with an epifluorescence microscope are used to calculate the concentration of peptide as a function of distance, with the mucus buffer interface located 100 μm from the origin. The images and concentration profile shown are of the positively charged peptide, 900 s after the beginning of the experiment. The gray, shaded area between the buffer and mucus curves is a graphical representation of the metric (Methods).

Charge is one parameter commonly considered to influence transport through mucus, since the interactions of a particle with surrounding matrix components are mediated by surface-to-surface contacts. Consequently, we generated two short peptides with uniform charge: the positively charged “+” peptide consists of 10 lysine residues, each separated by one alanine residue, while the negatively charged “−” peptide consists of glutamic acid residues separated by alanines (Fig. 3a). All peptides were covalently linked to the fluorescent dye FAM (6-carboxyfluorescein). This fluorescent tag is small, singly charged, and is not expected to affect transport into or through mucus.

We quantified partitioning and transport of the reporter peptides through mucus using a previously described microfluidics device^21, 38^. With this setup, a continuous aqueous flow of fluorescent peptides is generated within a micron-scale channel at carefully controlled rates adjacent to a stable mucus layer. The small sample volume (5 μL) required for this system is ideally suited for the study of native mucus samples, which are typically not available in large volumes. The peptide solutes partition into the mucus layer, and their subsequent transport is monitored with fluorescence microscopy (Fig. 3a and b). The rate of entry into the mucus barrier, as well as diffusion and peptide-mucin binding interactions within the mucus, result in peptide concentration profiles that can be quantified.

**Figure 4:**
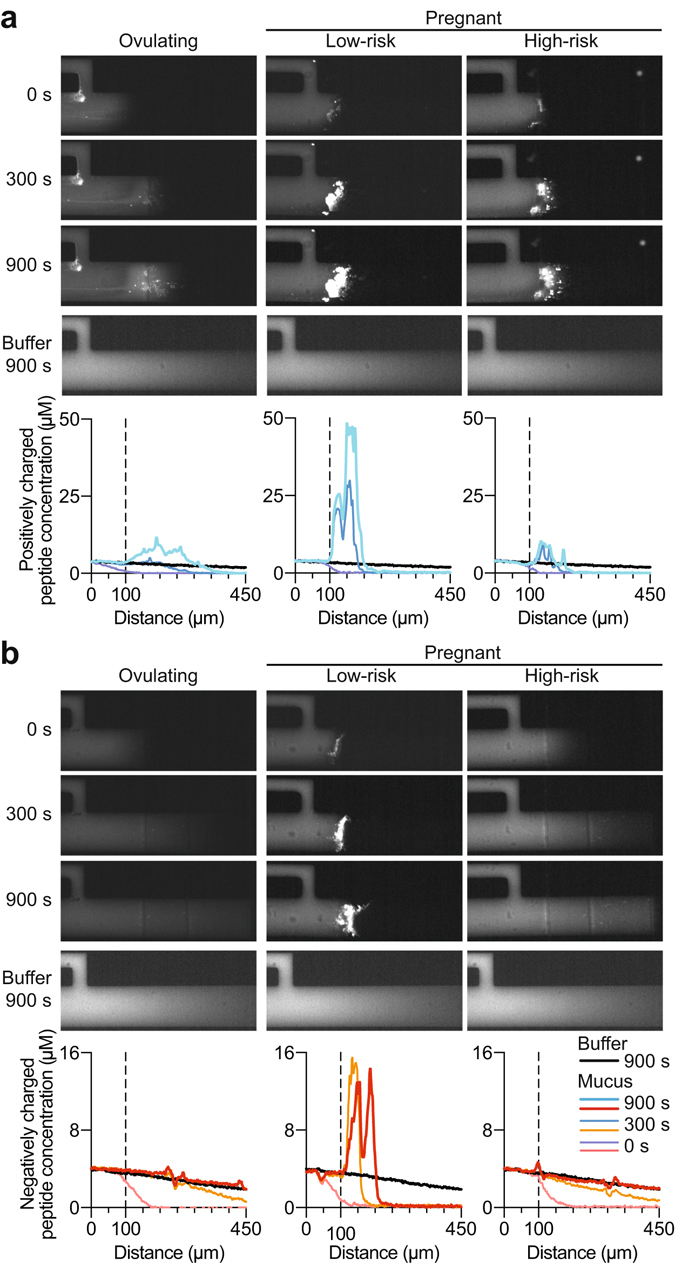
Peptide diffusion through cervical mucus reveals differences in permeability and mucoadhesion between ovulating non-pregnant, low-risk, and high-risk pregnant patients. Microscope images and respective concentration profiles from a representative single patient from each of the three patient groups are shown. (**a**) Positively charged peptides (top panel, blue) enrich substantially more at the interface of mucus from pregnant patients in contrast to ovulating non-pregnant patients, indicating increased mucoadhesion. However, this enrichment is decreased in mucus from high-risk patients. (**b**) Negatively charged peptides (bottom panel, red) diffuse freely through mucus from ovulating and high-risk pregnant patients, but not through low-risk patient mucus, indicating increased adhesiveness of mucus from healthy pregnant patients.

**Figure 5:**
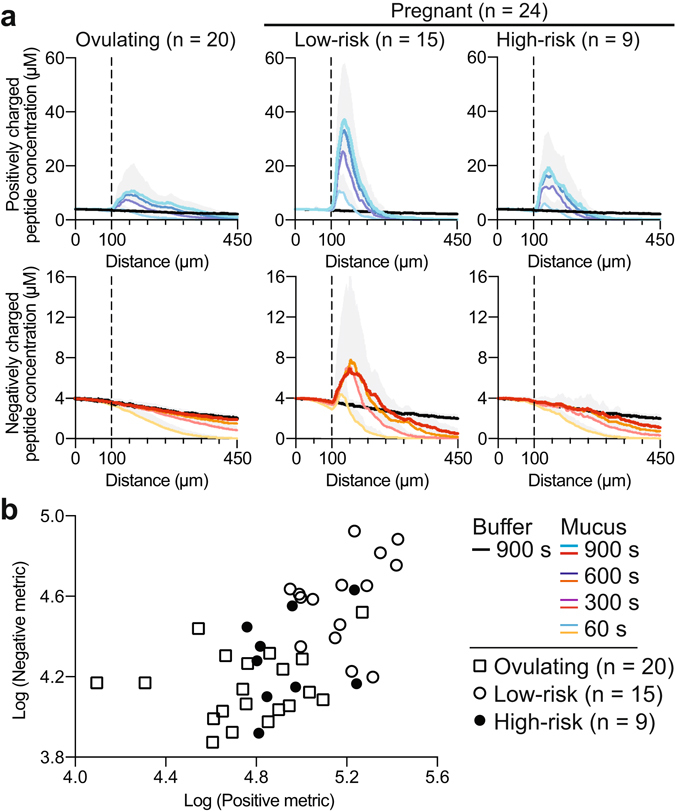
Peptide diffusion through cervical mucus enables stratification of patients at high and low risk for preterm birth. (**a**) Average concentration profiles of positively charged peptides (top panel, blue) show significantly increased adhesiveness in pregnant patients; however, the enrichment of positively charged peptides in mucus from high-risk patients is significantly lower compared to that of low-risk patients. Negatively charged peptide concentration profiles (bottom panel, red) differentiate between high- and low-risk patients, indicated by free diffusion through mucus from ovulating and high-risk patients, but not through mucus from low-risk patients. In all concentration profiles, the vertical, dotted line indicates the buffer-mucus interface. (**b**) Scatter plot of log-transformed negative and positive peptide permeability metric values reveals that high-risk patient mucus is more permeable to peptide probes. Metric values represent the difference in peptide transport behavior between mucus and buffer, integrated over the length of the channel and duration of the experiment.

We quantified the transport of (+) and (−) charged peptides in mucus from 20 ovulating, 15 low-risk pregnant, and 9 high-risk pregnant patients (Table 2; see the table caption for specific information on cohort composition). As in the SPT experiments, all pregnant patients were gestational-age matched at the time of collection (*P* = 0.07, Student’s t-test); however, high-risk patients gave birth preterm (*P* = 1.31E-4, Student’s t-test). Peptides at a concentration of 4 μM were introduced into the microfluidic channel in the presence of the mucus sample or in the presence of buffer. At this concentration, the peptides do not significantly affect the ionic environment of the mucus layer. Concentration profiles of the fluorescent peptides were acquired through the center cross - section of the mucus channel (Fig. 3a,b, Methods). Representative examples of fluorescence images of channels filled with mucus from three patients, recorded 0 s, 300 s, and 900 s after introduction of the (+) peptide and (−) peptide, appear in Fig. 4a and b, respectively. The corresponding profiles quantifiying the peptide concentration as a function of distance through the channel are shown below each set of images. We detected a peak in the (+) peptide concentration profile at the mucus-buffer interface for all sample categories (Figs 4a and 5a). This peak is indicative of enrichment, which is essentially an increase in local concentration of peptide at the interface of the mucus barrier. This enrichment is likely due to upward partitioning consistent with electrostatic interactions with the negatively charged mucins, as well as possible direct binding interactions between the peptides and mucins that enhance the local peptide concentration. Even though all sample categories showed enrichment, the degree to which they were enriched differed substantially. The average maximum concentration of the (+) peptide that accumulated inside mucus from ovulating women was 10.6 (SD ± 9.7) μM (Fig. 5a). For comparison, the same peptide accumulated to substantially higher levels in mucus from women at low risk for preterm birth, where the maximum concentration reached 36.9 (SD ± 20.9) μM (Fig. 5a). Samples from high-risk pregnant patients had levels of enrichment (19.3 (SD ± 13.2) μM) that were more typical of ovulating women (Fig. 5a

**Table 2:** Patient characteristics associated with samples of cervical mucus samples used in peptide permeability measurements.

Characteristics	Non-pregnant (n = 20)	Pregnant (n = 24)		p-value
	Ovulatory (n = 20)	Low risk (n = 15)	High risk (n = 9)	
Age (years)	35.40 (±3.69)	27.80 (±5.47)	24.67 (±6.40)	
Race (%)				
White	100.00	60.00	44.44	
Black	0.00	0.00	11.11	
Hispanic	0.00	33.33	33.33	
Other	0.00	6.67	11.11	
HIstory (%)				
Nullipara	70.00	0.00	0.00	
Primipara	0.00	53.33	77.78	
Multipara	30.00	46.67	22.22	
Prior PTB	0.00	0.00	0.00	
Gestational Age (weeks)				
Collection	NA	31.66 (±1.88)	29.43 (±3.08)	0.07
Delivery	NA	39.96 (±0.60)	33.59 (±2.85)	1.31E-04
Positive GBS (%)	NA	26.67	22.22	
Dilation (cm)	NA	0.0 (±0.0)	3.1 (±1.2)	5.54E-05

Values represented as mean (±standard deviation) or percent (%) where applicable.

).

For the (−) peptide, in ovulating women (Figs 4b and 5a), a decreasing peptide concentration gradient through the mucus layer was observed; this gradient was nearly identical with or without mucus present (Fig. 5a), demonstrating a lack of interactions between mucus and the (−) peptide. For comparison, in mucus from low-risk pregnant patients, we consistently found a slower evolution of the flow profile, resulting in substantially lower concentrations (0.50 (SD ± 0.47) μM) of the peptide at the end of the channel (450 μm) at equivalent measurement times (Fig. 5a). This effect could be related to reduced diffusivity or downward partitioning of the (−) peptide in the mucus. This phenomenon did not occur in mucus from high-risk pregnant patients (1.10 (SD ± 0.53) μM), where the peptide appeared to diffuse through mucus nearly as effectively as in ovulating women (Fig. 5a). In addition, in several, but not all, low-risk pregnant individuals, we observed weak enrichment of the negative peptide at the mucus barrier interface, reflecting peptide retention by the mucus components. Retention of the peptide at the interface was not detected in any of the high-risk samples measured (Fig. 5a). Two conclusions can be drawn from this experiment. First, the microscopic permeability of mucus from ovulating, pregnant, and high-risk women differ significantly and can be assessed with short, charged peptides, with each charge revealing a unique binding profile characteristic of a specific mucus category. Second, the more similar permeability behavior toward both (−) and (+) peptides of mucus from high-risk pregnant patients and ovulating patients versus their low-risk pregnant counterparts strongly suggests that the barrier properties of cervical mucus are compromised in cases of elevated risk of preterm birth.

Having seen the qualitative differences in peptide transport between subject groups (Figs 4a,b and 5a), we sought to test the statistical significance of these results. A quantitative metric was devised for measuring the difference in permeability of each peptide by integrating the difference between the concentration profile in buffer and in mucus over space and time. Briefly, the metric is the difference in peptide concentration between a channel filled with buffer and a channel filled with mucus, integrated over the entire length of the channel in every image taken every 10 s for the duration of the 15-min experiment (Methods, Eq. 4; Fig. 3b, integrated area between the concentration curve of peptide in mucus and buffer, highlighted in gray). Integration over the entire experiment accounts for the integrated area calculated from each concentration curve measured at each time point. Log-transformed metrics for positive and negative peptides in each patient mucus sample are summarized as a scatter plot in Fig. 5b. The increased enrichment and decreased penetration depth of both peptide types in mucus from low-risk patients, for example, results in higher metric values (Figs 4a and 5b, Methods). Conversely, peptide diffusion behavior in mucus that mimics that observed in buffer yields a lower metric value (Figs 4a and 5b, Methods). We performed Hotelling’s T-square tests to determine the statistical significance of the permeability differences among the three patient groups. Considering both peptides in the test, the permeability of mucus from pregnant patients was significantly reduced in comparison to ovulating non-pregnant patients (*P* = 7.1E-6, unequal variance Hotelling’s T-square test). Further, mucus from high-risk patients was significantly more permeable to both peptides than mucus from low-risk patients (*P* = 5.3E-3, unequal variance Hotelling’s T-square test). The metrics calculated for mucus from low-risk individuals clustered at higher values (Fig. 5b). These data confirmed the hypothesis that charge-selective mucus permeability as measured by a set of judiciously designed peptides is a robust indicator of altered physiology of the mucus barrier.

Our permeability assays and SPT experiments revealed that cervical mucus from our three patient groups restricted the passage of charged, micron-sized particles and nanoscale peptides in significantly different ways. At the microscopic length scale where steric interactions dictate permeability, probe particles were significantly more mobile in ovulatory mucus than samples from pregnant patients. Although on average particles were more mobile in cervical mucus from high-risk than low-risk patients, these results are not significant (Fig. 1c). The absence of statistical significance between the high- and low-risk patient groups using this technique suggests that structural reorganization alone cannot fully account for the significant rheological differences measured previously between these two patient groups^7^, and that biochemical modifications to the mucins may contribute to these differences. Indeed, we found that cervical mucus from low-risk patients was significantly more effective at excluding both negatively and positively charged nanoscale peptides (Figs 4b and 5a), presumably due to repulsive electrostatic interactions in the case of the former and electrostatically mediated binding to mucus polyanions in the case of the latter.

This study highlights that cervical mucus from pregnant women at risk for preterm birth is more permeable than that from women with healthy pregnancies. The intimate relationship between hydrogel permeability and network physicochemical properties renders this finding clinically relevant, as it suggests that risk for preterm birth is associated with altered retention of host immunological molecules and interaction of microbes with the cervical mucus barrier. In previous work we reported that mucus from high-risk patients is more translucent and has a higher spinnbarkeit than samples from low-risk patients, leading to the hypothesis that cervical mucus in high-risk patients fails to develop into the thickened and impermeable barrier typically present during healthy pregnancies^7^. The results of the current investigation support this hypothesis and additionally show that mucus from high-risk patients is more “leaky” than cervical mucus in healthy pregnancy (Fig. 5a and b).

Our finding of significantly increased mobility of nanoscale peptides, (Fig. 5a and b) but not micron-sized tracer beads (Fig. 2a and b) in cervical mucus from high-risk patients suggests that this increased permeability is primarily attributable to altered adhesiveness of the components of the mucus barrier. In previous work, we showed that mucin polymers alone can regulate the diffusion of small peptides and even protons^21, 38^, and hence we speculate that altered mucin biochemistry can impact overall mucus permeability. As a result, it can be expected that the regulatory role of the cervical mucus barrier in selectively restricting or permitting the passage of particles and microbes may be compromised through alterations to the biochemical and/or adhesive properties of the constituent mucin molecules. Mucins interact with and potentially regulate the activity of a plethora of non-mucin proteins in mucus, particularly ones involved in innate host defense^39^. For instance, our data predict that host antimicrobial peptides, which are generally highly cationic and contribute antimicrobial properties to the cervical mucus plug^5, 40^, would be less well retained in high-risk cervical mucus that is more permeable to positively charged peptides, thus making infection more common in this patient group. Modified mucoadhesive and biochemical properties of mucin molecules may also affect the movement of viral particles in cervical mucus^29^, and likely also regulate the physiology of microbes^41–43^.

The altered mucoadhesive properties of the cervical mucus in high-risk women may be due to modification of mucin glycosylation patterns. One possible mechanism for this may be the pathogenic cleavage of sialic acid residues from mucin glycans, which could simultaneously alter the charge structure of the mucin molecules in patients at higher risk for bacterial infection as well as increase the flexibility of the mucin chains. This is consistent with our observations of reduced positively charged peptide interactions and the elevated spinnbarkeit measured in high-risk samples^7^. Additionally, bacterial vaginosis, a condition in which the normal vaginal flora are dominated by the pathogens *Gardnerella vaginalis* and *Mycoplasma hominis*, is associated with increased mucinase and sialidase activity in cervico-vaginal mucus secretions^44–46^ and significantly associated with intra-amniotic infection and incidence of preterm birth^18, 47–50^. While it remains uncertain whether the changes observed in cervical mucus are a cause or a consequence of preterm labor, our findings clearly demonstrate that the biophysical and biochemical properties of cervical mucus from women who deliver preterm are significantly different from those who carry to term. Future longitudinal studies will aim to elucidate the specific physicochemical modifications to the mucin molecules that underlie these effects as they occur throughout pregnancy.

At present, there is a critical clinical need for predictive diagnostic tools and biomarkers for preterm birth. Current routine diagnostic risk screenings include measurement of cervical length, and although the correlation between a short cervix and preterm birth is significant, the positive predictive value of a short cervix for preterm delivery is low^8, 51–56^. Additional known risk factors include the patient’s history of preterm birth and increased fetal fibronectin levels^56–58^, but many preterm births occur in women without risk factors, and many women who are identified as high-risk deliver at term, highlighting the need for reliable diagnostic biomarkers. Our finding that the permeability of cervical mucus is correlated with pregnancy risk may be exploited as a powerful diagnostic resource because of the accessibility of this biological material. Our hope is that mucus permeability could be developed into a routine test to assess preterm birth risk, and help establish therapeutic interventions for high-risk women, such as a synthetic mucus mimetic that could curb bacterial ascension by reproducing the healthy physicochemical properties of the cervical mucus barrier that are lacking in patients at high risk for preterm birth.

To conclude, the results from this study in combination with our previous rheological findings^7^ establish the potential of cervical mucus as a sensitive diagnostic tool for disease. These findings may help in the development of improved, targeted treatments by spotlighting the particular importance of reinforcing the cervical mucus plug in cases of compromised barrier function. More broadly, we expect that the identification of general biophysical patterns and the underlying biochemical changes that influence and modulate the selective barrier properties of mucus will be essential for improving our mechanistic understanding of not only preterm birth but mucosal diseases as a whole, whether they affect the cervix, lungs, gastrointestinal tract, or any surface of the body where mucus is found.

## Materials and Methods

### Mucus sample collection

#### Ovulating patients

Our study included 20 ovulating non-pregnant individuals aged 18–45 years, with no restrictions based on race, ethnicity, or spoken language. Our exclusion criteria were recent sexually transmitted infection, intercourse within 24 h of collection, abnormal PAP smear within the last 6 months, or cervical surgery within the last 6 months. Patients with polycystic ovarian syndrome were excluded from the current study. Patients receiving treatment for infertility were excluded. Ovulation was detected via self-administration of a urine luteinizing hormone test kit (recommended kit: Clear Blue Easy, SPD Swiss Precision Diagnostics GmbH, Switzerland) prior to a procedure (i.e., intrauterine insemination). The number of days of the menstrual cycles did not define “ovulation”: the window of “ovulation” for collecting ovulatory mucus was defined as 36 h from a positive ovulation result. After informed consent was obtained from 20 patients, specimens were collected during a sterile speculum exam with a 1-mL insulin syringe immediately before any other procedure. Specimens were gathered directly from the external cervical os after clearing the area of vaginal discharge. Vaginal discharge, if present, was removed by wiping with a large-tip swab (Scopette). Cervical mucus was not removed by this process because cervical mucus is adherent to the cervical canal. Mucus was immediately snap-frozen in liquid nitrogen and stored at −80 °C prior to analysis.

#### Pregnant patients

We enrolled pregnant women (18–50 years old) with singleton pregnancies at gestational ages of 24–34 weeks. We enrolled two patient groups: (i) 22 healthy, low-risk women being followed in an outpatient obstetric clinic, and (ii) 16 high-risk women admitted to the hospital following symptomatic preterm labor, defined as cervical dilation of at least 1 cm in the setting of uterine contractions. High-risk patients were invited to participate after contractions had stopped and only when no cervical exam had been performed for 48 h. Patients were not in active labor at the time of enrollment, but were in a state of arrested preterm labor. High-risk patients who gave birth after 37 weeks gestation (n = 7) as well as low-risk patients who gave birth prior to 37 weeks gestation (n = 2) were not included in the results of this study.

Pregnant patients were excluded for the following reasons: maternal medical conditions predisposing the patient to preterm delivery, placenta previa, active vaginal infection at the time of sample collection, pelvic exam or sexual intercourse within 48 h of sample collection, and the presence of labor, rupture of membranes, or active vaginal bleeding at the time of sample collection. Pregnant patients receiving intra-muscular or vaginal progesterone doses were also excluded.

Cervical mucus was sampled via sterile speculum examination. Prior to mucus collection, the area surrounding the external cervical os was cleared of vaginal discharge. If vaginal discharge was present on the cervical surface, then it was removed as for ovulating patients. A sterile catheter 3.1 mm in diameter (Aspirette Endocervical Aspirator, Cooper Surgical, Trumbull, CT, USA) was placed at the external cervical os and used to aspirate a distal specimen of cervical mucus. If cervical mucus was visible, then a cervical cytobrush was used to collect the specimen. Mucus samples were snap-frozen in liquid nitrogen and stored at −80 °C prior to analysis.

Out of concern for the welfare of our patients, care was taken to remove the minimum amount of mucus necessary for this study. Two previous studies reported the average weights of the entire cervical mucus plug from pregnant women after term delivery to be 7.0 g (n = 15)^59^ and 6.87 g (n = 18)^6^. Whole plugs from women who delivered preterm weighed an average of 3.8 g (n = 4)^59^. In our cohort, on average, the estimated weight of mucus collected was 134 mg (±180 mg) from low-risk pregnant patients (n = 14) and 316 mg (±207 mg) from high-risk pregnant patients (n = 9). We estimate the average fraction (by weight) of extracted mucus to be 2–8% of the entire plug.

### Mucus sample preparation and storage

Samples were assigned sequential numbers for cataloging according to the date of collection. Researchers were not blinded to group allocation when performing each experiment and assessing the outcome, due to the visible macroscopic differences between samples in each patient category. For example, spinnbarkeit can readily be observed during pipetting of the samples or during injection of the samples into the capillaries and microfluidic devices. Additionally, nearly every cervical mucus sample from non-pregnant ovulating women was optically clear, in contrast to the mucus from pregnant women, which was typically opaque.

All samples were delivered frozen to MIT. Upon receipt, samples were divided into aliquots of various masses to reduce the number of freeze-thaw cycles. Samples were stored at −80 °C until use. Aliquots were thawed immediately prior to use. All samples experienced no more than two freeze-thaw cycles before use, thus normalizing the effects of freezing over all samples. The microstructure of mucus appears affected beyond two freeze-thaw cycles (Fig. S1a), while the adhesive properties of mucus appear less sensitive to repeated freezing/thawing (Fig. S1b).

### Single particle tracking (SPT) experiments and analysis

#### Experimental protocol

SPT specimens were prepared by combining 15 μL of cervical mucus with 0.5 μL of a solution of fluorescent, negatively charged (carboxylated) microspheres 1 μm in diameter (Magsphere, Inc., Pasadena, CA, USA) in deionized water at a dilution ratio of 1:400, resulting in an overall dilution ratio of 1:12,000 for the microspheres. If more or less than 15 μL of mucus were available, then the volume of bead solution was adjusted to accommodate a 30:1 (mucus:microsphere solution) volume ratio. All specimens were subsequently vortexed for 30 s to ensure adequate mixing, then pipetted into borosilicate square capillaries 0.9 mm × 0.9 mm × 15 mm (#8290; Vitrocom, Mountain Lakes, NJ, USA). Capillaries were sealed on both ends using a 1:1:1 mixture of petroleum jelly, lanolin, and paraffin, and then mounted onto microscope slides for imaging.

Imaging was performed at 30.3 frames per second for 10 s and at room temperature with a Zeiss Axio Observer D.1 inverted microscope using a Zeiss LD Plan-Neofluar 20x/0.4 Corr Ph2 objective lens (Carl Zeiss Microscopy GmbH, Jena, Germany) and a Hamamatsu Flash 4.0 C11440–22CU camera (Hamamatsu Photonics, Hamamatsu City, Japan).

#### Analysis

An average of 150 particles were imaged for each specimen from an average of eight movies recorded at different locations within the glass capillaries. For each image frame (Fig. 1a), particles were identified using publicly available MATLAB (v8.2.0.701; Natick, MA, USA) code^60, 61^, which identifies candidate features using high-intensity matches and filters them using criteria such as maximum feature eccentricity and radius of gyration^60^. The x and y position of each approved particle in each frame is recorded by the same code as the center of mass of the localized image intensity. As a result of the apparent drift in many of the trajectories, we applied a drift-correction algorithm taken from publicly available microrheology software^61^, which subtracts the center-of-mass motion of all particles in a given frame from each individual trajectory. However, significant amounts of drift remained after application of this code as a result of the non-uniformity of this relaxation process, which caused displacements of various degrees throughout the sample. When we performed de-drifting over each individual particle as opposed to using the center-of-mass motion of the entire frame (Fig. S2 in the Supporting Material), the ballistic motion at large delay times was largely eliminated. However, this technique is not reliable at large delay times, since it imposes a net zero displacement for every particle at all delay times, as evidenced by the plateauing or sudden change in the MSD slope at large values of τ (Fig. S2b). Further, the MSDs at early lag times (τ < 0.3s) from which the fits and statistical comparisons between patient groups were constructed were relatively unaffected by the choice of de-drifting algorithm, as confirmed by the similar fit values obtained for *α* and *D*_*α*_ for all patient groups and the invariance of the conclusions regarding statistical significance (Fig. S3 in the Supporting Material). As a result, de-drifting using the center of mass of each frame was applied for the analysis reported in the main text of this manuscript, since it did not lead to this artifact at larger lag times.

From these drift-corrected x and y positions, the time-averaged MSD for a sequence of *N* images for the *k* ^th^ particle is^20, 34^

3 $$\overline{{r}_{k}^{2}(\tau )}=\frac{1}{N{-}^{\tau }/{\rm{\Delta }}t}\sum _{i=1}^{N{-}^{\tau }/{\rm{\Delta }}t}[{(x(i{\rm{\Delta }}t+\tau )-x(i{\rm{\Delta }}t))}^{2}+{(y(i{\rm{\Delta }}t+\tau )-y(i{\rm{\Delta }}t))}^{2}],$$

where ${\rm{\Delta }}t$ is the time between successive frames (Fig. 1b). The ensemble average over all *K* particles is^31^

4 $$ < {r}^{2}(\tau ) > =\frac{1}{K}\sum _{k=1}^{K}\overline{{r}_{k}^{2}(\tau ).}$$

Reproducibility of SPT results was qualitatively confirmed by comparing results in individual aliquots of mucus from individual patients (Fig. S4 in the Supporting Material).

Statistical analysis was performed using Prism v7.0 (Graphpad Software, Inc., CA). Grubb’s test for individual outliers was performed on log-transformed MSD values (at $\tau =0.1s$) extracted from each patient group (α = 0.05, *P* < 0.05; Fig. 1c, starred data). Grubb’s test assumes a normal distribution of data; therefore, MSD values were log-transformed. The diffusion behavior of particles in samples of mucus from two patients (Fig. 1c, starred) was abnormally separated from the other patients in each group: the value of the generalized diffusion coefficient *D*_*α*_ at early lag times in the sample from the pregnant patient (Fig. 1c, blue) was 3 orders of magnitude higher than the average value in mucus from all other pregnant patients. The value of *D*_*α*_ at early lag times in the sample from the ovulating patient (Fig. 1c, red) was more than one order of magnitude larger than the average value in mucus from all other ovulating patients. Thus, these two patients were removed from further analysis. The Mann-Whitney test for statistically significant differences between patient groups was used to accommodate non-normal distributions of values of *D*_*α*_ and *α*. Non-normality was determined using quartile-quartile plots of the data (Fig. S5 in the Supporting Material)

### Peptide diffusion experiments and analysis

#### Permeability-reporting peptides

Peptides were synthesized, purified, and identified by the Swanson Biotechnology Center at the Koch Institute at MIT (Cambridge, MA, USA). Two peptides, AK_10_ ((AK)_10_-NH_2_) and AE_10_ ((AE)_10_-NH_2_), were synthesized via 9H-fluoren-9-ylmethoxycarbonyl (FMOC) peptide synthesis and labeled with one 6-carboxyfluorescein at the N-terminus after synthesis. Peptides were purified using reverse-phase high performance liquid chromatography. Peptide identity and purity was confirmed via matrix-assisted laser desorption ionization mass spectrometry. Before use in the microfluidic device, peptides were dissolved in H7 buffer (20 mM HEPES, 20 mM NaCl, pH 7) at a final concentration of 4 μM.

#### Experimental protocol

Microfluidic polydimethylsiloxane devices were designed and fabricated as previously described^21, 38^, and cured at 95 °C for 48–72 h prior to use. Devices bonded to microscope slides were mounted on an inverted epifluorescence microscope (IX-71, Olympus American, Central Valley, PA; or Zeiss Observer Z1) equipped with a 5x or 10x objective and LED light source or mercury lamp (excitation at 450–495 nm or 475 nm, respectively). All channels were washed with H7 buffer before use. The two valves were closed or opened by applying or releasing pressure with a 3-mL syringe connected to the valves via polytetrafluoroethylene tubing. All experiments were carried out at room temperature. One to two microliters of sample were injected to fill the main channel. The two valves were closed while the inlet and outlet channels were washed with H7 buffer. The top valve was opened, exposing the mucus interface. Twenty-five microliters of peptide solution were added to the reservoir of each inlet. The flow rate of the peptide solution into the inlet channel was determined by gravitational flow. An image of the channels was captured every 10 s for 20 min. At least two successful experimental replicates were obtained for each patient mucus sample, except for one sample of cervical mucus, for which only one measurement was obtained.

#### Analysis

Images were analyzed using ImageJ (v1.47; Wayne Rasband, National Institutes of Health, Bethesda, MD, USA; http://imagej.nih.gov/ij) and MATLAB software. The zero time point was defined as the first image when the fluorescently labeled peptide passed the inlet-outlet branch point. The distance x-axis parameter origin represents a point in the buffer solution 100 μm before the estimated mucus-buffer interface. If no mucus-buffer interface existed, as in control H7 buffer experiments, then the zero distance point was assigned to the edge of the initial wave of fluorophore at 0 s. Data were excluded if: i) the mucus sample swelled out of the main channel; ii) the mucus-buffer interface became eroded or moved; or iii) the bottom valve broke during the 15-min timeframe. Four cervical mucus samples from pregnant patients were excluded because of the first criterion. Transport curves were generated by measuring the fluorescence intensity of each pixel in a 20 pixel-wide lane along the central axis of the mucus channel. Peptide concentration was calculated from fluorescence intensity as

5 $$Peptide\,concentration\,(\mu {\rm{M}})=c(x)=(\frac{{I}_{F}-BKG}{{I}_{Bath}-BKG})\ast \,4\,\mu M,$$

where fluorescence intensity (*I*_*F*_) is the average fluorescence intensity per unit length along the axis of the channel from the origin located 100 μm before the buffer-mucus interface to the end of the channel (Fig. 2). Bath intensity (*I*_*Bath*_) is a 10 × 10 pixel region in the washed outlet channel beyond the mucus interface, which is assumed to be equivalent to the intensity of a 4 μM solution of the peptide in buffer. The background intensity (*BKG*) is a 10 × 10 pixel region not occupied by a channel.

For each type of peptide, the concentration in buffer at each point, *x*, along the channel axis was averaged over all runs to provide a buffer concentration function *c*_*buffer*_(*x,t*), where *x* = 0 at 100 μm before the mucus-buffer interface, collected at 10-s intervals up to 15 min. Intensity profiles for each sample run gave a function *c*_*sample*_(*x,t*), also at 10-s intervals for 15 min. The concentration profile of peptide in buffer was subtracted from that of the same peptide type in a given mucus sample and integrated over the entire 450 μm distance (Fig. 2b, 900 s, the shaded area between concentration profiles of positively charged peptides in mucus and buffer). These profiles were integrated over the 15-min experiment in 10-s intervals. The diffusion metric *M* was calculated as

6 $$M={\int }_{0}^{900}{\int }_{0}^{450}|{c}_{sample}(x)-{c}_{buffer}(x)|dx\,dt.$$

The upper integration limit of 450 μm was chosen to be slightly lower than the minimum profile width in all the samples. Numerical integration of the discrete intensity profiles was performed using the trapezoidal rule. The final *M* value for a given sample and peptide was the average *M* over all technical replicates. The number of channels evaluated per patient varied between 2 and 17 replicates, based on the amount of available mucus and the presence of excluding behavior, like swelling, within the microfluidic device. The mucus from one patient could only be measured in singlet. Quantitative analysis was performed using MATLAB. Reproducibility was confirmed by measuring the permeability of two separate aliquots of mucus from a single patient in two separate batches of microfluidic devices (*P* = 0.0085; Fig. S6 in the Supporting Material). In this case, the metric values were summed to create a single metric.

Statistical analysis was performed using the Real Statistics Resource Pack software (Release 4.3; Charles Zaiontz, www.real-statistics.com). Metric values were log-transformed to improve normality of the distributions (Fig. S7 in the Supporting Material). Significance was calculated using Hotelling’s T-Square test with unequal variance to accommodate negative and positive metrics.

### Study approval

The study protocol was approved by the Institutional Review Boards of Tufts Medical Center (#9355), the Women and Infants’ Hospital of Rhode Island (WIH 15–0073), and The Massachusetts Institute of Technology (#1501006840R001). Written informed consent was obtained from all participants prior to enrollment in the study. All experiments were performed in accordance with the relevant guidelines and regulations.

## Electronic supplementary material

Supplementary Information
