# Supplementary material for: Probing the potential of mucus permeability to signify preterm birth risk
Source: Sci Rep. 2017 Sep 4;7:10302. doi: 10.1038/s41598-017-08057-z (PMC5583328; doi:10.1038/s41598-017-08057-z)
Supplement: Supplementary file 1 — Supplementary Information [file 41598_2017_8057_MOESM1_ESM.pdf]

Probing the potential of mucus permeability to signify preterm birth risk

K. B. Smith-Dupont, C. E. Wagner, J. Witten, K. Conroy, H. Rudoltz, K. Pagidas, V. Snegovskikh, M. House, K. Ribbeck*

SUPPLEMENTAL FIGURE LEGENDS

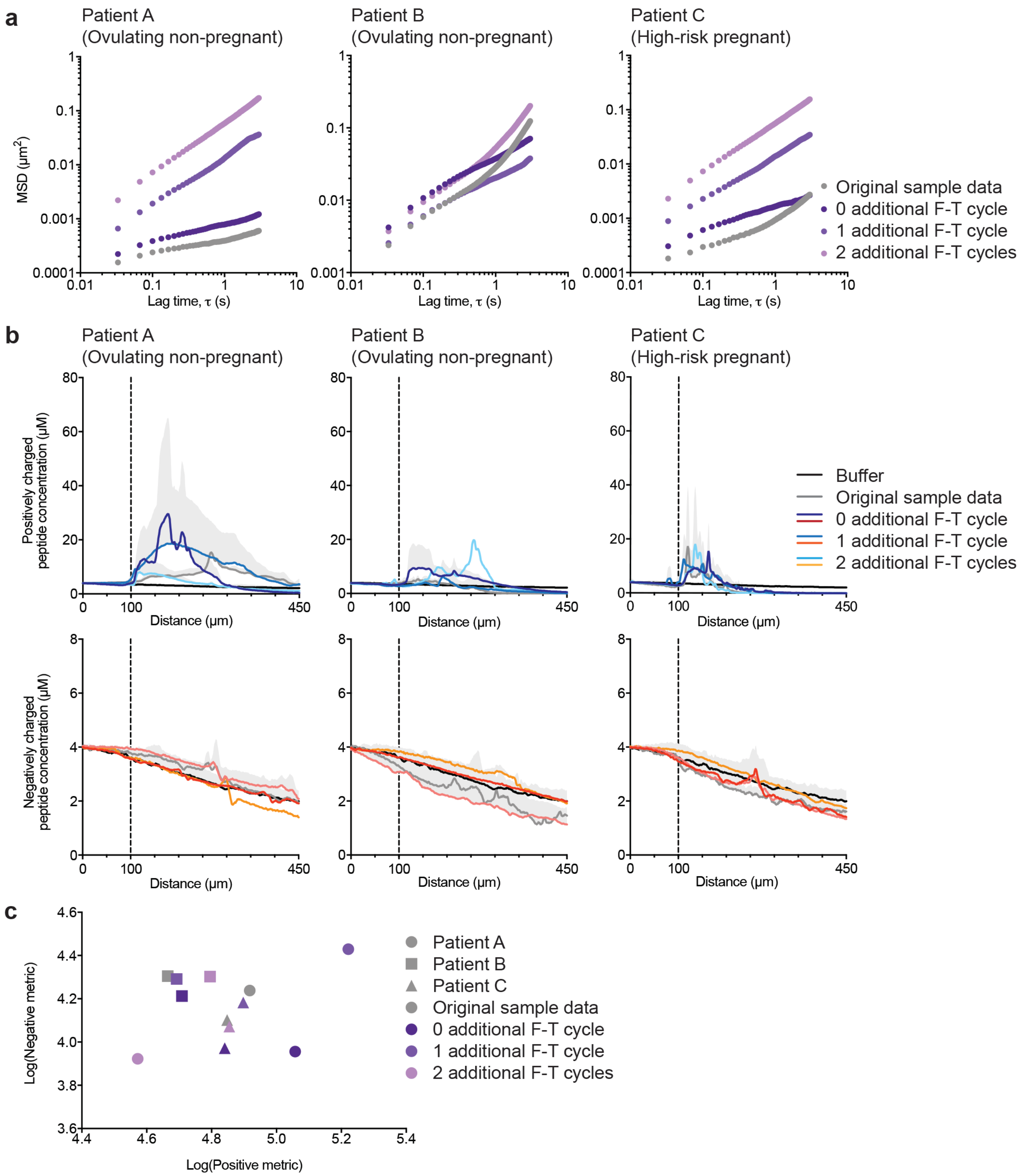

Supplemental Figure S1. Repeated freeze-thaw (F-T) cycles affect the microstructure of cervical mucus and possibly its adhesiveness. To determine the impact of F-T cycles on mucus integrity, permeability was measured in three cervical mucus samples across three cycles. F-T cycles were performed by snap-freezing the sample in liquid nitrogen, and then thawing it at 4 °C for as many

cycles as indicated. (**a**) MSD of particles in mucus from three individuals after additional F-T cycles reveals an increase in the mobility of the micron-sized tracer particles as a function of number of F-T cycles in mucus from two patients (A and C), but not the third (B). Data presented in the main body of the manuscript for each individual patient are shown in grey. (**b**) Average concentration profiles of positively (top, blue) and negatively (bottom, red) charged peptides through mucus from three individuals show no drastic change in the permeability of either peptide with number of F-T cycles for patients B and C, while data gathered from the mucus of patient A were more variable between cycles. Peptide diffusion profile in buffer is shown in black, and data presented in the main body of the manuscript for each individual patient are shown in grey. In all concentration profiles, the vertical, dotted lines indicate the buffer-mucus interface. (**c**) Scatter plot of metric values confirms consistent peptide diffusion behavior across F-T cycles in mucus from patients B and C, but more heterogeneity is observed in mucus from patient A. Metric values represent the difference in peptide transport between mucus and buffer, integrated over the channel length and experiment duration.

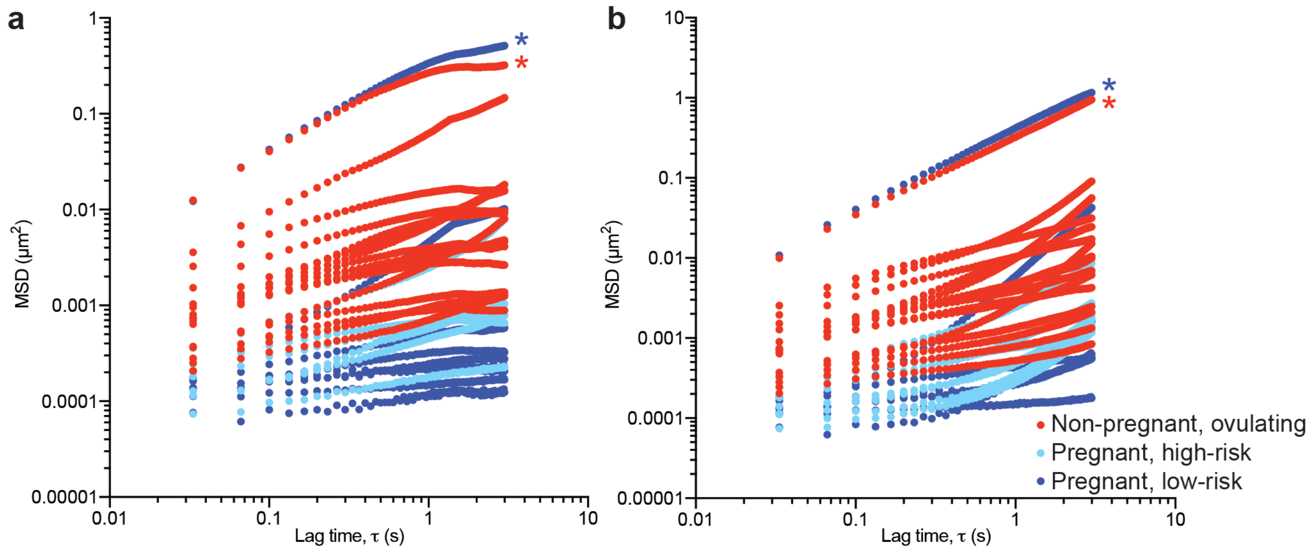

Supplemental Figure S2. MSD of particles de-drifted with the (**a**) individual particle algorithm introduces anomalies at large delay times in comparison to the (**b**) center-of-mass algorithm.

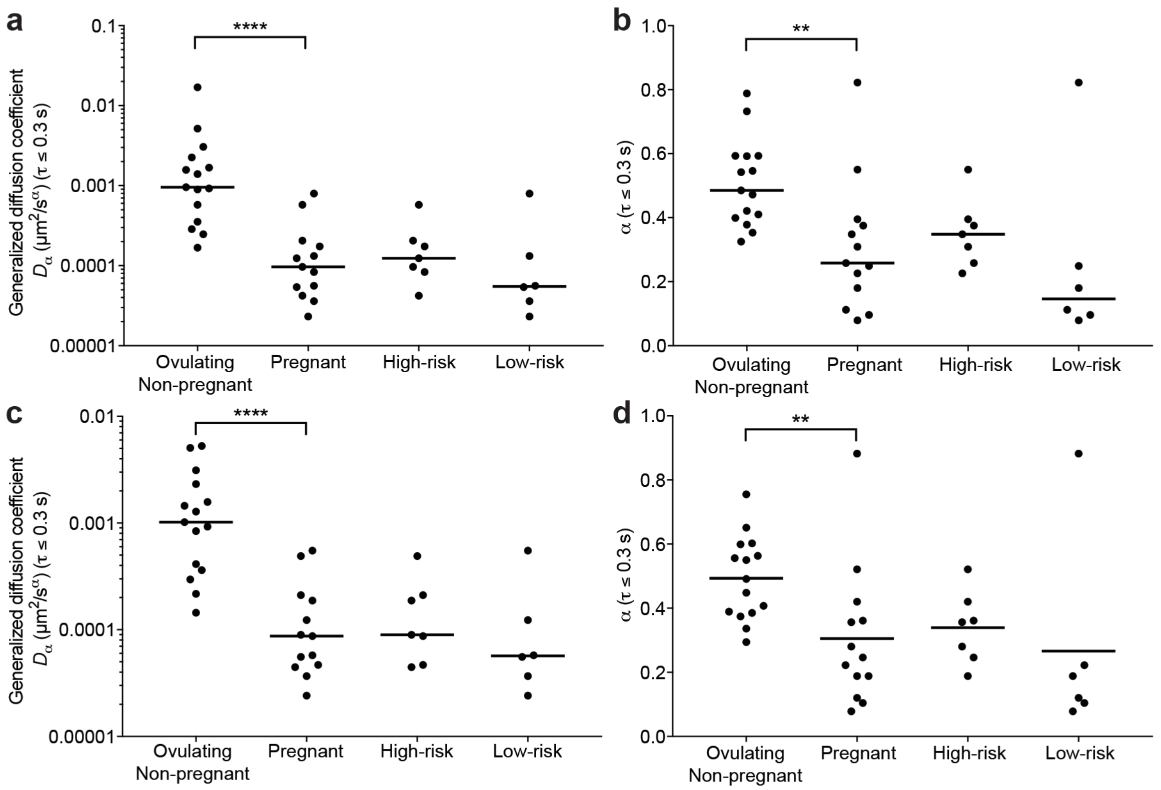

Supplemental Figure S3. Significance of restricted particle mobility statistics (generalized diffusion coefficient, D_α, and (subdiffusion exponent, α) in mucus from pregnant patients is unaffected by de-drifting algorithm (**a** and **b**: individual particle, **c** and **d**: center-of-mass). (**a**) Diffusivity (D_α) and (**b**) subdiffusion exponent α values extracted from MSD results at early lag times (≤ 0.3 s). Each point represents a single patient sample from non-pregnant ovulating (n=16) or pregnant (n=14) patients. Pregnant patient data are further subdivided into high (n=7)- and low (n=7) -risk groups. Bars, median values for each patient group. Significance was determined with the Mann-Whitney test (****P<0.0001; **P<0.0015). (**c**) Diffusivity (D_α) and (**d**) subdiffusion exponent α extracted from MSD results at early lag times (≤ 0.3 s). Each point represents a single patient sample from non-pregnant ovulating or pregnant patients. Pregnant patient data are further subdivided into high- and low-risk groups. Bars, median value for each patient group. Significance was determined with the Mann-Whitney test (****P<0.0001; **P<0.0021).

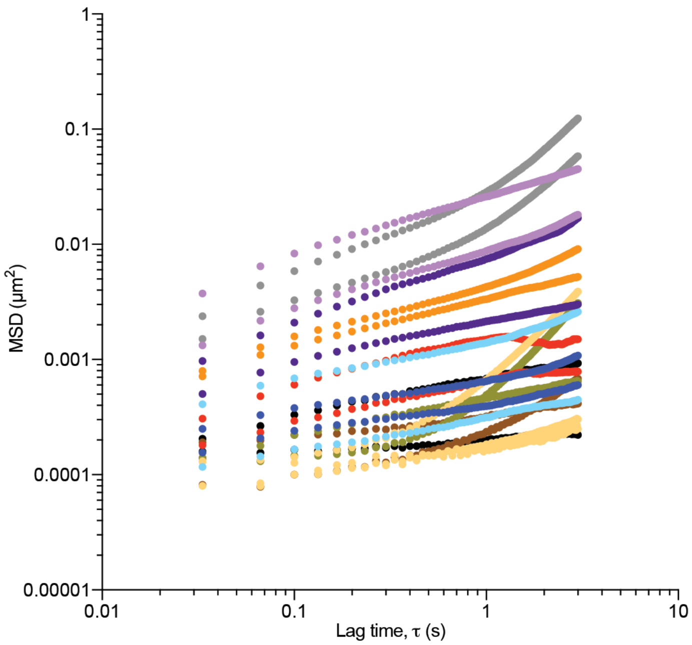

Supplemental Figure S4. MSD curve from particle tracking in separate aliquots of cervical mucus from an individual patient is reproducible. 2 or 3 separate aliquots of cervical mucus from 11 patients were measured. Measurements from the same patient are paired by matching colors.

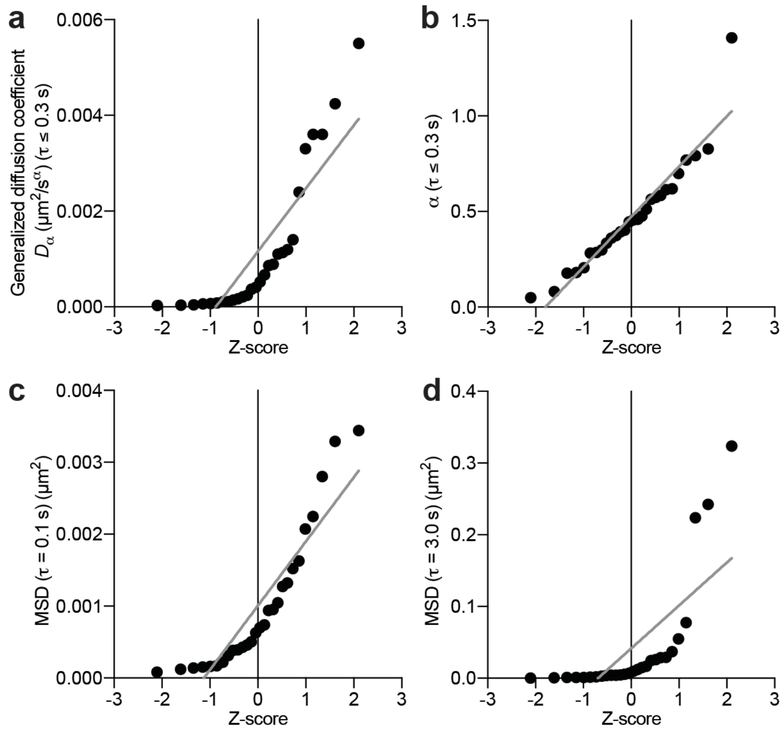

Supplemental Figure S5. Parameters extracted from Single Particle Tracking (SPT) microrheology experiments in cervical mucus are not normally distributed. Quartile-Quartile (QQ) plots of Diffusivity, *D*, and anomalous diffusion exponent, α, extracted at early lag times (**a** and **b**). (**c** and **d**) QQ plots of MSD values at 0.1 s and 3.0 s lag times, respectively.

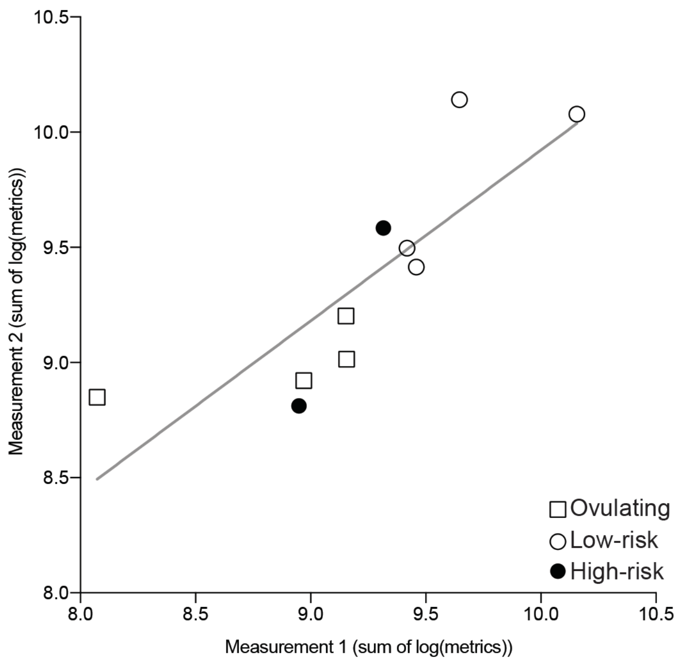

Supplemental Figure S6. Permeability metric calculation from separate aliquots of mucus from an individual patient is reproducible. The sums of the positive and negative metrics taken from two separate measurements of mucus from separate aliquots form 10 patients are plotted as a function of one another. The grey curve represents the best-fit line, and confirms positive correlation between the measurements (P = 8.5E-3).

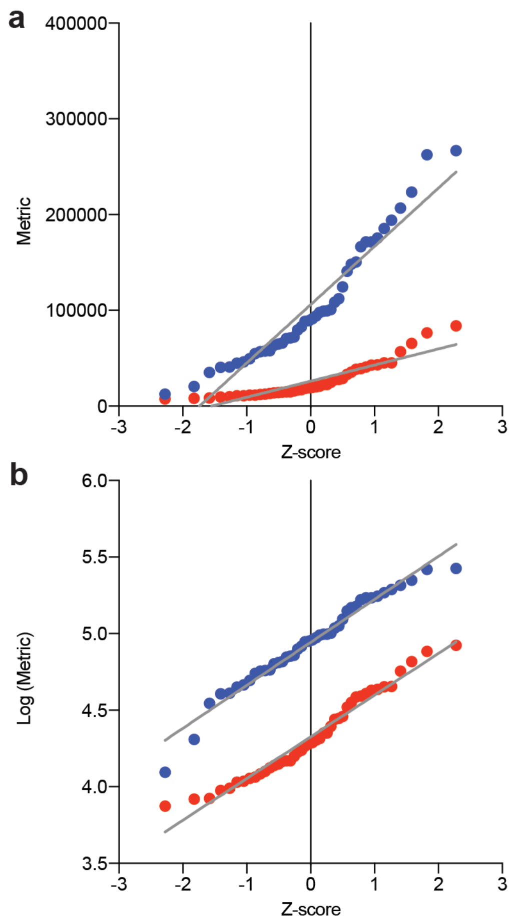

Supplemental Figure S7. Permeability metric values are only normally distributed upon log-transformation. Quartile-Quartile (QQ) plots of raw (**a**) and log-transformed (**b**) permeability metric values. Metric values corresponding to positive and negative peptides are represented as blue and red spheres, respectively. Grey curves represent best-fit lines to the data.
